# Plasmodium falciparum molecular surveillance to inform the Mozambican National Malaria Control Programme strategy: protocol

**DOI:** 10.1136/bmjopen-2024-092590

**Published:** 2024-11-24

**Authors:** Clemente da Silva, Gloria Matambisso, Simone Boene, Eduard Rovira-Vallbona, Arnau Pujol, Kiba Comiche, Antoni Sánchez, Bryan Greenhouse, Arlindo Chidimatembue, Andrés Aranda-Díaz, Paulo Arnaldo, Cristina Ariani, Patrick Walker, Henriques Mbeve, Nelo Ndimande, Dário Tembisse, Shazia Ruybal-Pesántez, Robert Verity, Bernardete Rafael, Baltazar Candrinho, Alfredo Mayor

**Affiliations:** 1 Centro de Investigação em Saúde de Manhiça (CISM), Manhiça, Mozambique; 2 ISGlobal, Barcelona, Spain; 3 EPPIcenter Research Program, Division of HIV, Infectious Diseases, and Global Medicine, Department of Medicine, University of California San Francisco, San Francisco, California, USA; 4 Instituto Nacional de Saúde, Maputo, Mozambique; 5 Wellcome Sanger Institute, Hinxton, Cambridge, UK; 6 MRC Centre for Global Infectious Disease Analysis, Imperial College London, London, UK; 7 National Malaria Control Program, Ministry of Health, Maputo, Mozambique; 8 Facultat de Medicina i Ciències de la Salut, Universitat de Barcelona (UB), Barcelona, Spain; 9 Faculdade de Medicina, Universidade Eduardo Mondlane, Maputo, Mozambique; 10 Spanish Consortium for Research in Epidemiology and Public Health (CIBERESP), Madrid, Spain

**Keywords:** Malaria, Pregnancy, Epidemiology, PARASITOLOGY, Pregnant Women, Molecular biology

## Abstract

**Abstract:**

**Introduction:**

Malaria molecular surveillance has the potential to generate information on biological threats that compromise the effectiveness of antimalarial interventions. This study aims to streamline surveillance activities to inform the new strategic plan of the Mozambican National Malaria Control Programme (2023–2030) for malaria control and elimination.

**Methods and analyses:**

This prospective genomic surveillance study aims to generate *Plasmodium falciparum* genetic data to monitor diagnostic failures due to *pfhrp2/3* deletions and molecular markers of antimalarial drug resistance, to characterise transmission sources and to inform the implementation of new antimalarial approaches to be introduced in Mozambique (chemoprevention and child malaria vaccination). The study, to be conducted between 2024 and 2026, will use three sampling schemes: a multicluster probabilistic health facility survey in the 10 provinces of the country to detect *pfhrp2/3* deletions and markers of antimalarial drug resistance; dense sampling of all clinical cases in representative districts in the south targeted for elimination to characterise malaria importation and identify sources of transmission; and testing of pregnant women for malaria at their first antenatal care visit to assess malaria burden and molecular trends. Using a multiplex amplicon-based sequencing approach, the study will target microhaplotypes informative of genomic diversity and relatedness, as well as key drug resistance-associated genes, *pfhrp2/3* deletion and malaria vaccine targets. Key genomic information will be visualised in a dashboard integrated into the District Health Information System V.2-based Malaria Information Storage System for programmatic use.

**Ethics and dissemination:**

The protocol was reviewed and approved by the national ethics committee of Mozambique (Comité Nacional de Bioética para Saúde, Ref: 680/CNBS/23). Project results will be presented to all stakeholders using study-specific brochures and published in open-access journals.

**Trial registration number:**

NCT06529237.

**STRENGTHS AND LIMITATIONS OF THIS STUDY**

- A multicluster probabilistic health facility sampling scheme will be used to detect *pfhrp2/3* deletions and markers of antimalarial drug resistance across the 10 provinces in Mozambique.
- High-throughput sequencing will be used to target 165 microhaplotypes informative about *P. falciparum* genomic diversity and relatedness as well as markers associated with antimalarial drug resistance, diagnostic failure and vaccine targets.
- A new outbreak detector (EpiFRIenDs) will be used for outbreak monitoring and investigation of transmission sources in malaria elimination areas.
- Malaria incidence will be calculated considering potential biases (health-seeking behaviour, reporting and testing and alternative causes of fever) and used to compare its relationship with metrics of parasite genetic diversity.
- District Health Information System V.2-based genetic dashboard will be used for an easy visualisation of genomic outcomes.

## Introduction

 Mozambique is among the 10 countries with the highest malaria burden in the world, with an estimated 10.4 million cases in 2022.^1^ Malaria transmission is highly heterogeneous across the country, with a high burden in the north and a very low burden in the south, requiring different strategies for effective control and elimination.^2^ With the aim of strategically using genetic variation in *Plasmodium falciparum* to strengthen the decision-making capacity of malaria control and elimination programmes,^3^ a functional malaria molecular surveillance (MMS) system has been established in Mozambique^4^ to (1) detect the emergence of mutations conferring resistance to antimalarial drugs (ie, artemisinin-based therapies and sulfadoxine–pyrimethamine [SP] used for chemoprevention)^5^ and deletions affecting rapid diagnostic test (RDT) sensitivity (ie, *P. falciparum* histidine-rich protein 2 [*pfhrp2*])^6 7^; (2) characterise key drivers of ongoing malaria transmission with the goal of identifying transmission foci^8 9^ and differentiate between indigenous and imported cases in areas approaching elimination^10^,^12^; and (3) test the value of *P. falciparum* genetic data to recapitulate epidemiologic features of malaria transmission and inform the effectiveness of antimalarial interventions.^13^,^18^ As part of these activities, *P. falciparum* epidemiologic and genetic surveillance has also been integrated into antenatal care (ANC) clinics as a cost-effective sentinel surveillance approach.^19^,^21^

**Figure 1:**
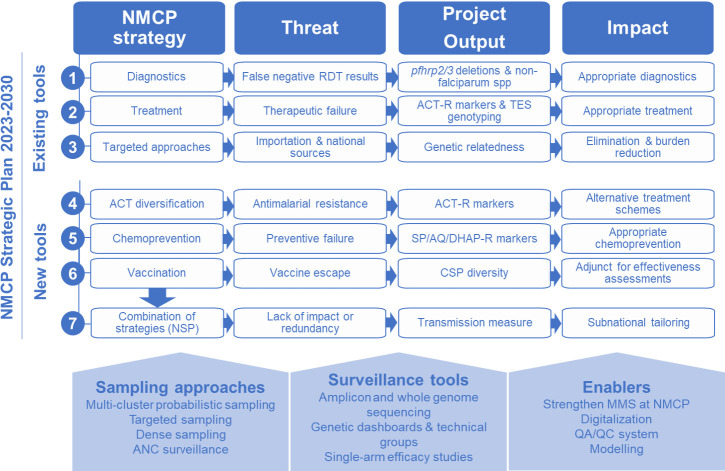
Project expected outputs and impact. ACT, artemisinin-based combination therapy; ACT-R, Resistance to ACT; ANC, antenatal care; CSP, ciscumsporozoite protein; MMS, malaria molecular surveillance; NMCP, National Malaria Control Programme; NSP, national strategic plan; QA/QC, quality assurance/quality control; RDT, rapid diagnostic test; SP/AQ/DHAp-R, Resistance to sulfadoxine-pyrimethamine/amodiaquine/dyhidroartemisinin-piperaquine; TES, therapeutic efficacy study.

The National Malaria Control Programme (NMCP) in Mozambique is entering a new strategic cycle (2023–2030) with a plan that includes genomic surveillance to guide programmatic decisions on five key antimalarial tools (figure 1): (1) malaria diagnosis using RDTs based on histidine-rich protein 2 (HRP2); (2) treatment with artemisinin-based combination therapies (ACTs), including diversification schemes to reduce the emergence of resistance^22^; (3) chemoprevention for pregnant women (intermittent preventive treatment in pregnancy), children (perennial malaria chemoprevention [PMC] and seasonal malaria chemoprevention [SMC]) and all populations in elimination and emergency settings (mass drug administration [MDA]); (4) introduction of the R21/Matrix-M vaccine^23^; and (5) reactive interventions in very low-transmission settings. However, as in most high-burden countries, limited resources make it difficult to roll out these interventions simultaneously throughout the country. In addition, there are constantly evolving threats to the effectiveness of these interventions, including gene deletions of RDT targets,^6^ infection with non-*falciparum* species, resistance to therapeutic^24 25^ and preventive^25 26^ antimalarials, importation of infections,^27^ immune escape to malaria vaccines (R21/Matrix-M^23^ and RTS,S^28^) as well as to other immunological interventions (eg, monoclonal antibodies^29^). Thus, the overall effectiveness of the NMCP strategy will depend on the NMCP’s ability to address emerging threats, prioritise protection for those most in need and deploy tools when and where they are likely to be most effective through local decision-making.

The goal of this project is to use MMS to optimise the public health benefits of the NMCP 2023–2030 strategy in Mozambique. The specific objectives are threefold. First, the project will enable real-time tracking of biological threats, including diagnostic failures due to *pfhrp2/3* deletions or non-*falciparum* infections, and assessment of molecular markers of first-line ACT resistance, supported by results from therapeutic efficacy studies (TESs). It will also characterise transmission sources at local and national levels through genetic classification of cases in low-transmission districts and identify national transmission sources and sinks. Second, the project will develop tools to guide decision-making on emerging threats to antimalarial approaches planned for use in Mozambique, including parasite resistance to chemopreventive drugs and vaccine escape^23 28^ and monitor intervention-driven changes in transmission using metrics of parasite genetic diversity.^30^ Third, the project aims to strengthen analytical and translational capacity to increase production throughput and uptake of MMS indicators to inform decision-making.

## Methods and analysis

### Study design and sample size calculations

**Figure 2:**
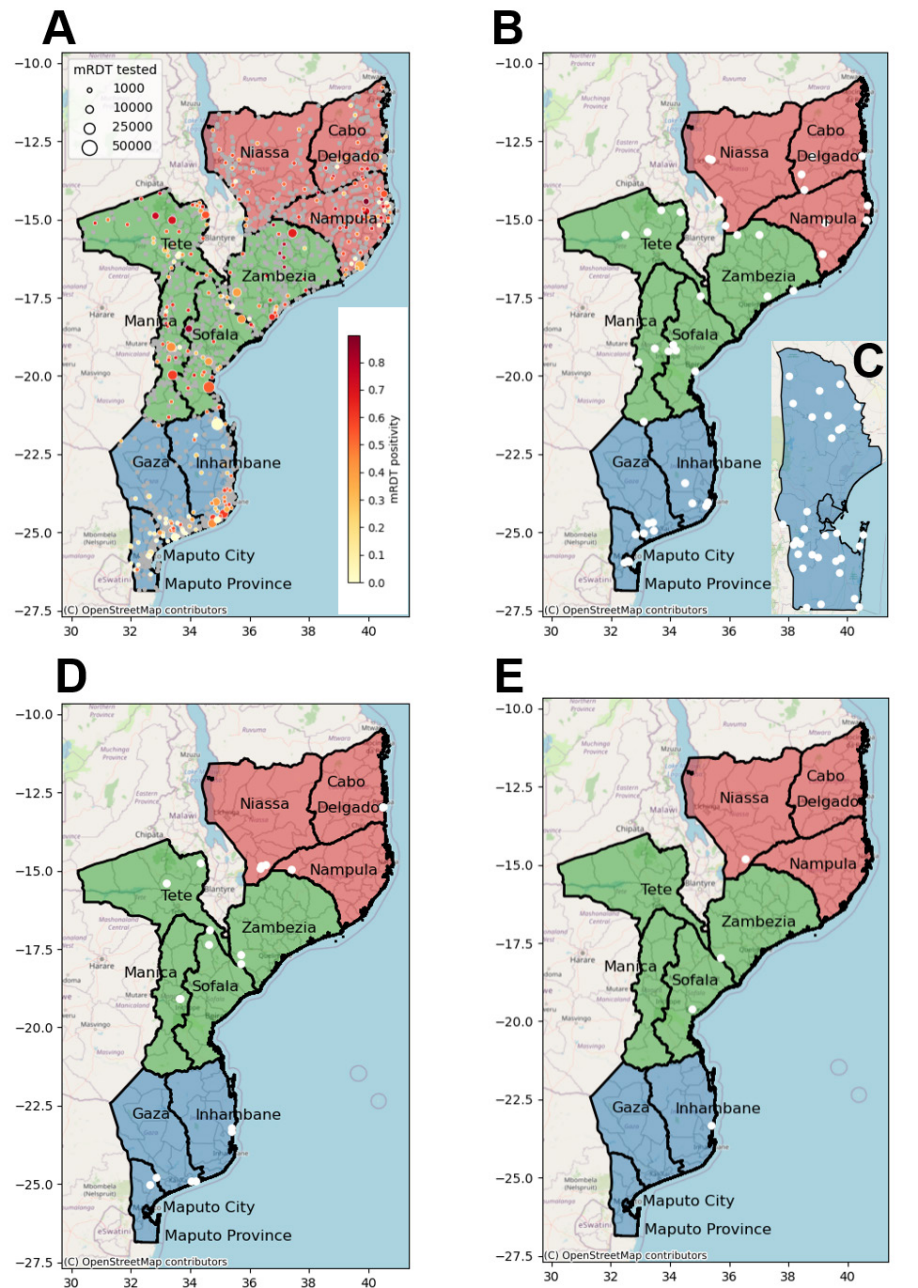
Map of the health facilities in Mozambique included in the malaria molecular surveillance project. (A) Health facilities in Mozambique. Mozambique is divided into 154 districts and 11 provinces (for surveillance purposes, we combine Maputo Province and Maputo city, giving a total of 10 provinces): three in the South region (in blue: Gaza, Inhambane, Maputo and Maputo city), four in the Central region (in green: Manica, Sofala, Tete and Zambezia) and three in the North region (in red: Cabo Delgado, Nampula and Niassa). The map shows Malaria rapid diagnostic test (mRDT) tested and *Plasmodium falciparum* positive cases among children aged 0–5 years in 2023 (District Health Information System V.2 data). The size and colours of the circle indicate the magnitude of mRDT tested and positive cases, respectively. Total number of health facility in Mozambique: 1517 (Cabo Delgado: 115; Gaza: 139; Inhambane: 134; Manica: 114; Maputo Cidade: 32; Maputo Province: 96; Nampula: 216; Niassa: 168; Sofala: 148; Tete: 131; Zambezia: 224). (B) Health clinics selected (white dots) for the multicluster sampling approach; (C) dense sampling (Maputo Province); (D) antenatal care clinics; and (E) sentinel sites where the therapeutic efficacy studies are conducted.

This is a prospective genomic surveillance study of *P. falciparum* samples to be collected between 2024 and 2026 from a variety of transmission intensities and geographies in Mozambique. Sampling design and efforts will be driven by target use cases. A multicluster probabilistic health facility sampling (HFS) sampling scheme^31^ will be followed to detect *pfhrp2/3* deletions and markers of antimalarial resistance across the 10 country provinces (figure 2A). Dried blood spots will be collected from a consecutive sampling of 60 RDT-confirmed malaria clinical cases per health facility, from a systematic random sample of four health facilities per province (figure 2B and table 1). Using the DRpower tool and associated web-based *pfhrp2/3* Planner,^32^ the study power was estimated to detect the prevalence of *hrp2/3* deletions above the 5% threshold at the regional level.^33^ Assuming an intracluster correlation coefficient (ICC) of 0.05 based on historical data (https://mrc-ide.github.io/DRpower/articles/historical_analysis.html), power was estimated at 92.4% (91.9%–92.9%) in regions with three provinces (12 clusters of 60 samples) and 96.9% (96.5–97.2%) in regions with four provinces (16 clusters of 60 samples). Power remains high under the more pessimistic assumption of ICC=0.1, being 78.6% (77.8%–79.4%) and 86.3% (85.6%–87%) for regions with 3 and 4 provinces, respectively. This same design will be used to estimate the prevalence of *pfdhps* mutations at codon 540 at the province level (margin of error analysis). Assuming a mutation prevalence of 90%,^34^ prevalence will be estimated to within 4.8% margin of error for ICC=0.01 and within 7.5% margin of error for ICC 0.05^31^ (assuming lower ICC than for *pfhrp2/3* given the lower variance between clusters expected when looking at smaller geographic scale). Finally, the design will be used to detect the presence of variants of concern (VOCs), namely *pfkelch13* markers of artemisinin partial resistance and *pfdhps-*581 marker of SP resistance, at the province level (presence/absence analysis). Assuming VOCs at 1% prevalence in the population, the design has 85% power to detect VOCs if ICC=0.01 and 67% if ICC=0.05^31^.

**Table 1:** Study provinces and districts targeted in the multi-cluster probabilistic health facility survey and antenatal care (ANC)-based sampling schemes

		Health facility (January–March 2025)		ANC		
Region	Province	District	Health facilities	District	Health facilities	Period
South	Maputo city	Kamavota	HC Mavalane			
		Kamubukwane	HC Bagamoio			
	Maputo Province	Manhica	HC Xinavane	Magude	HC Magude	January 2024–December 2025
		Matola	PH Matola		HC Motaze	January–December 2024
	Gaza	Bilene	HC Macia	Manjacaze	HC Chidenguele	January 2024–December 2025
		Chibuto	HC Mukhotwene		CHC Incadine	
		Chokwe	HC Xilembene			
		Limpopo	HC Chipenhe			
	Inhambane	Funhalouro	HC Mavume			
		Jangamo	HC Cumbana	Massinga	HC Massinga Sede	January 2024–December 2025
		Maxixe	HC A. Neto		HC Rio das Pedras	
		Panda	HC Panda			
Centre	Manica	Cid. Chimoio	HC 1 de Maio	Gondola	HC Josina Machel	January 2024–December 2025
		Gondola	HC Inchope		DH Gondola	
		Machaze	HC Sambassoca			
		Sussundenga	HC Rotanda			
	Sofala	Caia	HC Sena			
		Cid. Beira	HC Ponta Gea	Chemba	HC Mulima	January 2024–December 2025
		Gorongosa	HC Pungue		HC Chiramba	
		Nhamatanda	HC Metuchira Lomaco			
	Tete	Angónia	HC Ntsendeza	Angónia	HC Ntsendeza	September 2024–August 2025
		Chiúta	HC Manje	Chiúta	HC Manje	September 2024–August 2025
		Maravia	HC Chipera			
		Macanga	HC Gandali			
	Zambezia	Gurue	HC Cotxi	Mopeia	HC Mopeia sede	January 2024–December 2025
		Maganja da Costa	HC Nante		HC Gulamo	
		Molumbo	HC Molumbo			
		Pebane	HC 7 de Abril			
North	Nampula	Nampula	HC 25 de Setembro	Malema	HC Malema	January 2024–December 2025
		Ilha de Mocambique	HP Ampara			
		Moma	HC Chalaua			
		Nacala Porto	HC Nacala Porto			
	Niassa	Lichinga	HC Magga			
		Mandimba	HC Mandimba	Cuamba	HC Cuamba	January 2024–December 2025
		Mecanhelas	HC Muhala		HC Mepessene	
		Sanga	HC Nansinhenje		HC Titimane	
	Cabo Delgado	Balama	HC Kuekue			
		Cid. Pemba	HC Paquite	Pemba	HC Natite	September 2024–August 2025
		Montepuez	HC Mirate		HC Paquite	
		Namuno	HC Namacaca			

CidCidade (city)DHDistrict hospitalHCHealth centerHPHealth postPHProvintial hospital

A dense sampling approach, focusing on clinical cases from individuals>6 months of age, will be followed to characterise malaria importation and identify sources of transmission in representative districts in the south targeted for elimination (Magude, Matutuine and Namaacha districts in Maputo Province; figure 2C; table 2). This sampling will be implemented for 12 consecutive months, starting in April 2024 in Magude and Matutuine and in August 2024 in Namaacha. The selected areas, under the criteria of an average yearly incidence below five cases/week, include nine of the 11 health facilities in Namaacha, the 13 health facilities in Matutuine district and eight out of 10 health facilities from Magude district. Fifty samples will be collected from the four excluded health facilities in Magude (Magude sede and Motaze) and Namaacha (Mafuiane and Namaacha sede) to have a representative population of these four higher transmission areas. The total expected number of samples to be collected during 1 year is 290 in Magude, 630 in Matutuine and 550 in Namaacha.

**Table 2:** Districts and health facilities in Maputo Province targeted in the dense sampling scheme

District	Health facilities	Period
Magude	HC Capitine	April 2024–April 2025
	HC Facazissa	
	HC Mapulanguene	
	HC Panjane	
	HC Chichuco	
	HC Moine	
	HC Chicutso	
	HC Mahel	
	HC Magude	April 2024 until 50 samples collected
	HC Motaze	
Matutuine	HC Catuane	April 2024–April 2025
	HC Mabilibili	
	HC Mungazine	
	HC Ponta do Ouro	
	HC Zitundo	
	HC Gueveza	
	HC Manhangane	
	HC Ndelane	
	HC Salamanga	
	HC Hindanne	
	HC Matutuine	
	HC Nsime	
	HC Santa Maria	
Namaacha	HC Changalane	August 2024–August 2025
	HC Kulula	
	HC Matsequenha	
	HC Odete Mechisso	
	HC Dibinduane	
	HC Mundavene	
	HC Wamongo	
	HC Goba	
	HC Mahelane	
	HC Mafuiane	August 2024 until 50 samples collected
	HC Namaacha	

HChealth center

Pregnant women will be tested for *P. falciparum* malaria at their first ANC visit to assess malaria trends and MMS. During the first 2 years (January 2024 to December 2025), 1–3 ANC clinics per province will be selected (figure 2D; table 1) based on the average number of monthly recruitments in each province during 2023. Approximately 1300 samples are expected per each of the 10 provinces per year. Of the 13 000 samples collected across the country per year (total 26 000), 2870 are expected to be positive for *P. falciparum* by RDT (total 5740), based on the RDT-positivity rates obtained in 2023 at each province.

Finally, targeted sampling will be conducted at sentinel health facilities (figure 2E) in the four districts (Cuamba in Niassa, Mopeia in Zambézia, Dondo in Sofala and Massinga in Inhambane) where children aged 6–59 months (n=87 children per site) with microscopically confirmed uncomplicated *P. falciparum* malaria will be recruited for a TES.

### Enrolment of participants

HFSs will be conducted during the first trimester of 2025. Children aged 2–10 years with fever or history of fever (table 3) will be screened using routine HRP2-based RDTs (First Response Malaria Antigen *P. falciparum* HRP2 [Premier Medical] or SD Bioline Malaria Ag Pf, 05FK50 [Abbott,] depending on national procurement at the time of the study) and an additional confirmatory RDT targeting *P. falciparum* LDH (Biocredit Malaria AG Pf (pLDH) C14RHG25, Rapigen) selected from the recommended options in the WHO master protocol.^33^ Assuming that 85% of samples will be of high sequencing quality, the sample size per health facility will be increased from 60 to 71. Dried blood spots will be collected from study participants if either test is positive, and samples with discrepant RDT results will be used to detect *pfhrp2/3* deletions. The number of people to be screened at each health facility and the duration of recruitment to achieve the sample size will depend on the RDT positivity rate among study participants meeting the eligibility criteria.

**Table 3:** Study eligibility criteria

Inclusion criteria	Exclusion criteria
Children aged 2–10 years of age (cluster sampling). >6 months of age (dense sampling).	Any symptoms of severe malaria.
Fever (axillary temperature≥37.5°C) or history of fever in the preceding 24 hours.	
At least one positive parasitological test for malaria diagnosis via RDT*.	Negative parasitological test for malaria via RDT (except any women at their first ANC visit, who will be recruited before testing for malaria with an RDT).
OR	
Pregnant women (≥12 years of age) attending first antenatal care visit.	Unwilling to provide informed, written consent.
AND	
Informed, written consent to participate from participant and/or guardian.	History of antimalarial treatment in the last 14 days

* Routine one (First Response Malaria Antigen HRP2 [Premier Medical Inc] or Bioline Malaria Ag Pf, 05FK50 [Abbott], depending on national procurement at the tiem of the study) plus a second RDT (BIOCREDIT Malaria Ag pLDH), the latter being provided to support detection of deletions.Routine RDT (First Response® Malaria Antigen *P. falciparum* HRP2 [Premier Medical Inc] or SD Bioline Malaria Ag Pf [Abbott, 05FK50], depending on national procurement at the time of the study) plus a second confirmatory RDT (BIOCREDIT Malaria Ag Pf pLDH, Rapigen Inc), the latter being provided to support detection of *P. falciparum* parasites with *pfhrp2* deletions

ANCantenatal careRDTrapid diagnostic test

Women attending their first ANC visit (table 3) will be invited to participate in the study (if they live in the study area) by trained nurses, who will assign a unique identification number to consenting and eligible women and complete a brief form including date of visit, mother’s age, gravidity, week of pregnancy, area of residence and recent or past movements. Women will also be asked about health-seeking behaviours and use of interventions. Finally, women will be finger pricked for malaria RDT along with routine tests, treated if positive and a dried blood spot collected on filter paper for molecular analysis.

Dense sampling in Magude, Matutuine and Namaacha districts will be coordinated with district malaria focal points, community health workers (CHW), malaria volunteers (who provide a link between the CHW and the health facility, and assist the CHW in the follow-up of cases and administration of medication) and health facilities. All *P. falciparum* positive RDTs (SD Bioline Malaria Ag Pf, 05FK50, Abbott) among clinical cases (>6 months old; table 3) will be stored for molecular analysis. Once a case is notified, field workers will travel to the household of the case to collect Global Positioning System location within the period of 3 days (up to 7 days if needed).

### Data and sample collection

Field workers and nurses will be trained to ask for informed consent, perform a simple questionnaire and collect biological samples for molecular analysis.^4^ The survey questionnaire will be administered to all study participants or children’s parents/guardians meeting the inclusion criteria and will include characteristics of the participant and malaria related information. Nurses will be trained to collect blood by finger pricking following standard procedures.^4^ For each participant, either the *P. falciparum*-positive RDT used for routine malaria diagnosis (dense sampling scheme) or four blood spots onto two filter papers (Whatman 3 MM; all other sampling schemes) will be collected. Specimens will be labelled anonymously (patient unique identifier, study health facility and date), dried for 24 hours and kept in individual plastic bags with desiccants at 4°C. Every 2–6 weeks, the informed consents and samples will be sent to Centro de Investigação em Saúde de Manhiça (CISM) through a local transportation agency. A data manager will be responsible for the receipt of the informed consents, and a laboratory technician will be responsible for receiving the samples and storing them at −20°C until analysis. Part of the dried blood spot will be stored in RNA-preserving solution for future transcriptional studies. All samples will be kept in the CISM Laboratory for a period of approximately 15 years.

### Molecular analyses

We will genotype samples using targeted amplicon sequencing to characterise sequence (single-nucleotide polymorphisms [SNP], microhaplotypes and haplotypes) and copy number (duplications and deletions) variants. We will use the MAD^4^HatTeR targeted sequencing panels (developed at EPPIcenter—UCSF and Paragon Genomics, California, USA) on approximately 7690 *P*. *falciparum* samples (based on expected positivity rates). MAD^4^HatTeR is a modular assay that targets up to 239 *P*. *falciparum* amplicons of ≈250 bp, which include microhaplotypes informative about genomic diversity and relatedness in Africa,^19 35^ targets for *pfhrp2/3* deletions,^6 7^ *pfcsp* polymorphisms encompassing the TH2R and TH3R epitopes included in the R21/Matrix-M^23^ and RTS,S vaccine^28^ and drug resistance markers in 13 genes,^34^ including polymorphisms associated with resistance to artemisinin (*pfkelch13*),^36^ SP (*pfdhfr* and *pfdhps*),^37^ chloroquine (*pfcrt*^38^) and amodiaquine (*pfcrt* and *pfmdr1*). Additionally, targets for four non-*falciparum* species detection are included. Targeted amplicons obtained by PCR on genomic DNA using Illumina-specific adaptors and sample-specific barcode will be pooled to create a single product library, which will be sequenced (paired end 150 bp) on a MiSeq Illumina sequencer at CISM or higher performance equipment when available. We will use qPCR to determine parasite density,^39^ confirm *pfhrp2/3* deletions,^40^ *Plasmodium* species^41 42^ and copy-number variants (CNVs) in markers of piperaquine and mefloquine resistance (*pfpm2* and *pfmdr1*).^43^ We will also perform whole-genome sequence a subset of *P. falciparum* samples to generate a longitudinal catalogue of generate a longitudinal catalogue of genome-wide variations.

### Bioinformatic analysis

To infer alleles, FASTQ files from amplicon sequencing will be run through a Nextflow-based pipeline developed and benchmarked for data generated with MAD^4^HatTeR (https://github.com/EPPIcenter/mad4hatter). Briefly, primer dimers will be removed, and reads will be demultiplexed for each target, filtered based on quality and length, clustered using an error-inference model and aligned to the reference genome to remove non-specific amplification. To remove spurious variants in error-prone sequences, homopolymers and tandem repeats will be masked. Subsequently, low-abundance alleles (fewer reads than the maximum observed in negative controls or with within-sample allele frequency<1%), and samples with low target coverage (<50 targets for genetic diversity and relatedness with a read depth of>100) will be removed.^19^ Multilocus markers will be analysed using downstream modules for SNP phasing and multilocus allele frequency inference algorithms currently under development and benchmarking. We will identify putative CNVs using a generalised additive model to normalise read depth and calculate fold change across several targets for each gene of interest (*pfpm2* and *pfmdr1*). Putative CNVs will be confirmed by qPCR.^43^

### Quality control and assurance

The questionnaires will be administered to study participants using a Redcap tablet by public health technicians assigned to the health facilities by the Ministry of Health. At each site, a local supervisor will conduct a thorough review of the information before it is sent weekly to the data centre at the CISM. A quality control of the collected data will be performed weekly by data managers and research assistants, who interact with the local supervisors to correct possible errors.

A quality control programme for molecular determinations will be followed using an artificial set of controls with known genotypes (laboratory clones in monoclonal and polyclonal mixtures at different proportions and parasite densities). In addition, the team will participate in the WHO/United Kingdom National External Quality Assessment Service (UK NEQAS) scheme for molecular assays for *Plasmodium* species identification and *pfhrp2/3* gene deletion detection.

### Data management

**Figure 3:**
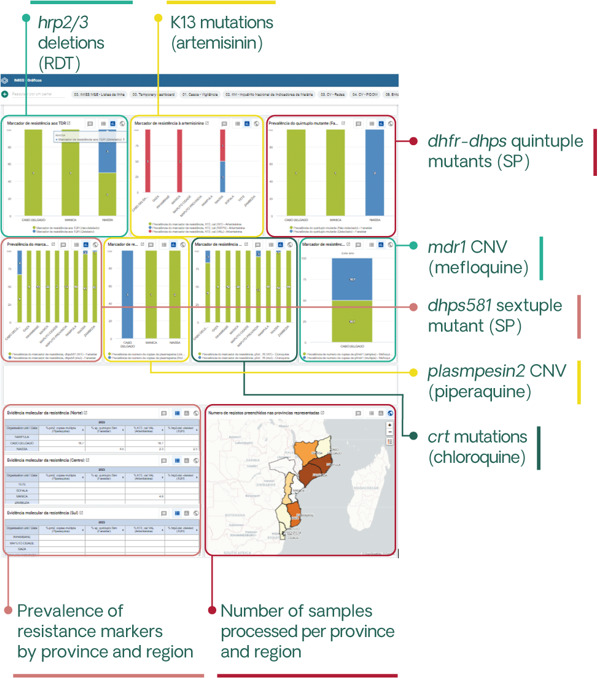
Genetic dashboard included in the District Health Information System V.2-based Integrated Malaria Information Storage System (iMISS) to facilitate data accessibility and use. The iMISS, which is currently being rolled out, includes case data, focus investigations, entomological surveillance data and intervention data. A genetic dashboard has already been created into IMISS corresponding to antimalarial resistance and *pfhrp2/3* deletion profiles, developed in collaboration with National Malaria Control Programme. Specific dashboards for genetic connectivity and case classification in very low-transmission areas (estimated proportion of imported cases, together with travel history and other parameters obtained from case-based notification tools) will be also considered. CNV, copy-number variant; RDT, rapid diagnostic test; SP, sulfadoxine–pyrimethamine.

Patient data will be collected using password-protected electronic devices. Automated quality checks will be performed to ensure data completeness. Confidentiality and security will be ensured through automatic encryption of sensitive data, storage on password-protected computers and in secured locations and data sharing using password-protected, encrypted files. Data will be deidentified prior to analysis, with the exception of geolocation codes required for spatial analyses. The study will also use data available from the NMCP, including District Health Information System V.2 (DHIS2) incidence data, intervention coverage, historical prevalence surveys, travel history or other mobility assessments and entomological data. Sequences generated from sample analysis will be integrated into a curated catalogue of genomic data, together with relevant anonymised clinical and epidemiological information, and made publicly available in public repositories such as the Sequence Read Archive (SRA, National Center for Biotechnology Information [NCBI]) or the European Nucleotide Archive, as well as the MalariaGEN Resource Center. To facilitate data accessibility and use by the NMCP and to achieve meaningful integration with other sources of surveillance data, genetic information will be incorporated into the DHIS2-based Integrated Malaria Information Storage System (iMISS), which is currently being implemented in Mozambique^44^ (figure 3).

### Study outcomes and analysis plan

This project has six primary endpoints. First, prevalence of drug-resistance markers at individual codons, or multicodon haplotypes if successfully phased, will be calculated at provincial level as the number of samples carrying a mutant allele or haplotype out of all samples with a valid genotype for the respective locus. Differences in the prevalence of drug resistance markers (1) between provinces; (2) between years and (3) after interventions will be evaluated using Fisher’s exact test. If emerging VOCs are identified, genetic relatedness between infections will be assessed to describe potential sources and origins of the variants. Allele frequencies will be estimated using methods that account for sample polyclonality.^45^

Second, the prevalence of suspected false-negative HRP2-RDT results among symptomatic patients with *P. falciparum* malaria will be determined at the regional level using the DRpower tool, which accounts for intracluster correlation when estimating the prevalence.^32^ The prevalence of false-negative HRP2-RDT caused by *pfhrp2/3* deletions will be determined as the number of *pfhrp2* or *pfhrp3* deletions confirmed by qPCR^40^ divided by the total number of confirmed cases in a given region.

Third, sources of importation and transmission will be identified using genetic relatedness (identity-by-descent [IBD]) between pairs of samples estimated using Dcifer software.^46^ The fraction of highly related pairs (using a threshold in IBD) will be calculated for samples within each province and between different provinces to characterise the spatial connectivity of parasite genetic populations. Isolation-by-distance will be calculated to assess the extent to which genetic relatedness decreases with geographic distance. Cases from areas targeted for elimination will be classified as local or imported infections by combining data on participants’ reported travel (dates and duration of travel), epidemiologic data from source and destination areas (malaria incidence estimated from DHIS2 data) and genetic data from source and destination areas (relatedness of samples via IBD). Outbreaks will be detected in the study areas using EpiFRIenDs software,^20^ and genetic relatedness analysis within outbreaks will be performed to quantify the number of transmission events causing the outbreak.

Fourth, genetic diversity metrics will be assessed to characterise natural and intervention-driven changes in transmission.^13^,^1647 48^ Correlation analysis will be used to test the relationship between parasite genetic diversity metrics (locus-based expected heterozygosity), within-host diversity data (multiplicity of infection [MOI] and effective MOI,^45^ Wright’s inbreeding coefficient, the proportion of polyclonal infections), the proportion of highly related samples based on IBD), RDT positivity rates at ANC clinics and DHIS2-based malaria incidence, the latter corrected for several potential biases (health-seeking behaviour, reporting and testing and alternative causes of fever).^1^ The use of microhaplotype-based genetic diversity markers to discriminate recrudescence from new infections in TESs will be compared with the currently recommended WHO/MMV *msp1*/*msp2*/*polyA*.^49^ In addition, the genetic diversity in the C-terminal region of the circumsporozoite protein that encompasses T-cell epitopes included in the R21/Matrix-M^23^ and RTS,S^28^ malaria vaccines will be determined by comparing the sequence reads with those in the *P. falciparum* 3D7 reference genome.

Finally, *P. falciparum* parasite rates and genomic indicators will be generated for the first ANC visit. RDT positivity rates and Insecticide-treated nets/indoor residual spraying coverage data will be compared with unbiased gold standard household-level data collected through the Malaria Indicator Survey. Time series analysis and logistic multivariate regression will be considered to assess seasonality and to estimate the change in parasite rates after introduction of antimalarial interventions, including an interaction term, area#timepoint, to identify effect differences among interventions introduced in the country (chemoprevention, malaria vaccine, vector control). A mechanistic transmission model, malariasimulation (https://mrc-ide.github.io/malariasimulation/), will be fitted to monthly ANC prevalence, adjusted for gravidity, using the siftr R package developed by Imperial College London to generate estimates of transmission and potential clinical case burden. Rates of polymorphisms associated with antimalarial, diagnostic and vaccine resistance will be compared in parasites collected from pregnant women and the general community.

### Ethics and dissemination

This study will be conducted in accordance with the ethical principles outlined in the Declaration of Helsinki. The protocol has been reviewed and approved by the institutional (CISM) and national ethics committee of Mozambique and the Hospital Clinic of Barcelona. Written informed consent will be obtained from all study participants prior to blood sampling. Two copies will be signed, one to be kept by the participants and the other by the investigators in a locked room. The information sheet and consent form will also include text explaining informed consent for future use of biological specimens for additional analysis of the *Plasmodium* parasite. In the case of minors (less than 18 years of age), consent will be obtained from parents, relatives or guardians. Informed consent will specify that anonymised data will be published. Enrolled participants will receive first-line antimalarial treatment in accordance with national treatment guidelines. There will be no economic incentive to participate in the study. Data and materials will only be transferred out of Mozambique if appropriate data and material transfer agreements are signed between the participating institutions.

## Discussion

Developments in genomics are driving some of today’s most groundbreaking research and surveillance approaches. However, the benefits of these tools will not be fully realised unless they are rigorously tested and deployed in an integrated manner across large geographic regions. The gap in the application and use of data and evidence generated by genomic surveillance is greater in Africa than in other parts of the world, despite a high burden of many infectious diseases, including malaria. As the archetype of a disease that primarily affects the Global South, malaria provides a unique opportunity to demonstrate how attention to equity in the deployment and use of genomics can realise its immense potential benefits for human health.

MMS activities have been designed for optimal sampling, with minimal bias and sample size to achieve appropriate statistical power for each of the use cases.^31^ The multicluster probabilistic health facility sampling (60 samples in four health facilities per province; 10 provinces) will ensure high power (>92%) at the regional level under typical ICC assumptions and good power (>78%) under pessimistic assumptions to detect *pfhrp2/3* deletions above 5%; a 5% margin of error to estimate *pfdhps*-540 prevalence at the provincial level under typical ICC assumptions and within 7.5% for pessimistic assumptions; and high power (85%) at the province level for typical ICC assumptions, although power drops (67%) for pessimistic assumptions, to detect VOCs. The collection of samples from all clinical cases (dense sampling) in representative districts in the South targeted for elimination will allow to quantify the overall impact of importation on sustaining transmission, as well as for outbreak monitoring. Finally, malaria surveillance at first ANC visits can provide information on malaria trends (including seasonal patterns to inform decisions on the introduction of interventions such as SMC) and a passive means of monitoring the impact of ongoing malaria interventions on transmission (and therefore delineate areas where cases are low because transmission has declined or where cases are low because interventions are highly effective) and sudden climatic changes on malaria burden.^20 21^ Blood samples from these pregnant women will be used for MMS to complement HFSs.^19^

Genomic surveillance data generated by these different sampling schemes will be integrated into several mathematical models, including a joint epidemiology–genetics model to infer sources of malaria transmission, burden stratification and impact assessment (Institute for Disease Modeling); a model linking malaria transmission in the general population to exposure to malaria in pregnancy^50 51^ (Imperial College) to infer near real-time patterns of malaria prevalence, transmission and burden from continuous ANC prevalence data; a deterministic multistrain model^52 53^ to quantify the protective efficacy of SP (PMC and SMC) against the major *pfdhfr/dhps* genotypes present in Mozambique (Imperial College and London School of Hygiene and Tropical Medicine); an individual-based malaria transmission model to assess the impact of diversifying ACT regimens (eg, multiple first-line therapies, geographic or target population stratification and ACT rotation) on the emergence of antimalarial drug resistance (Temple University)^54 55^; and a geospatial^56^ and landscape genetic model^57 58^ to identify key drivers of transmission (ISGlobal).

A multiplex sequencing panel (MAD4HatTeR) targeting up to 239 loci in the *P. falciparum* genome will be used for targeted amplicon sequencing.^19 35^ Amplicons will include microhaplotypes informative of genomic diversity and relatedness, as well as markers of resistance to the major antimalarial drugs used for treatment in Mozambique (*pfkelch13*, which mediates resistance to artemisinin^36^ and chemoprevention (*pfdhfr* and *pfdhps* genes, which mediate resistance to SP.^37^ This sequencing approach has been shown to be robust in detecting the high prevalence of *pfdhfr*/*dhps* mutations in Mozambique, but not *pfkelch13* mutations.^19^ In addition, microhaplotype-derived diversity metrics were shown to detect a decrease in parasite population size in an area targeted for malaria elimination,^19^ suggesting their value for assessing the impact of interventions. In addition, *P. falciparum* samples from pregnant women at their first ANC visit and children representing the community have shown consistent genetic diversity, interhost relatedness and prevalence of drug resistance markers, concluding the potential of pregnant women for molecular surveillance.^19^ Preliminary results also show that the microhaplotypes included in the panel allowed to detect a strong spatial differentiation of *P. falciparum* infection across Mozambique, highlighting the potential of malaria genomics for spatial analyses of malaria transmission. We will also evaluate the added value of using amplicon sequencing data for molecular correction in TESs compared with the currently recommended WHO/MMV *msp1*/*msp2*/polyA^49^ for differentiating recrudescence from new infections in TESs.

**Figure 4:**
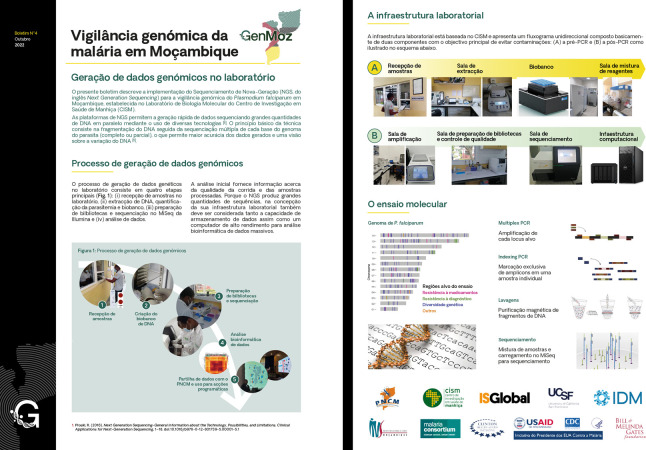
Example of a brochure produced for dissemination of the project activities to national stakeholders.

This project, guided by programmatic priorities and based on collaborative efforts, aims to promote the use of MMS indicators for decision-making. Data will be shared with the NMCP through biannual meetings and workshops to train in the interpretation of genomic data. A brochure in Portuguese will be produced every 4 months to present the main results of the project and shared with stakeholders in the surveillance field (figure 4; online supplemental appendix 1). Genetic data will be included in the DHIS2-based iMISS (figure 3) to facilitate data accessibility and use. The project will also support clinical trials in producing key outcomes (PCR-corrected efficacy in therapeutic efficacy trials) and in assessing the impact of interventions on the development of malaria resistance (PMC, SMC and MDA). Finally, the project will contribute to a curated sequence catalogue of targeted and whole-genome sequences that started with samples collected in 2015, which will be used for studying the evolution of *P. falciparum* parasites in response to changing national malaria control and elimination policies.

Purchase of reagents and equipment, international transferences, custom clearance and shipments are important and lengthy processes that must be handled to conduct molecular studies in Mozambique, as in other African countries. Due to the scarcity of local suppliers of reagents and sequencing consumables, imports become necessary, increasing the costs and project time. There is also uncertainty on the exact price of equipment and reagents due to exchange rate and import costs. To address this and other issues related with the implementation of the project activities, a project management plan will be developed to ensure that the activities are conducted timely and with quality. Monthly management meetings will monitor financial and resource allocation and a joint tracking document will facilitate these efforts. Communication and collaboration between stakeholders will be promoted through monthly meetings to provide updates and strategic adjustments. A monitoring and evaluation plan will be developed to ensure timely and comprehensive project tracking. Trimestral assessments will monitor results, with a shared folder for stakeholder access. Molecular methodologies will be transferred to the National Institute of Health (INS) in Mozambique, with the aim of strengthening the genomics workforce in Mozambique for public health action. Finally, the project will promote gender equity by highlighting the contributions of Mozambican women scientists through visually appealing posters distributed in educational and public spaces and dissemination activities during the International Day for Women and Girls in Science.

We expect that the genomic intelligence developed through this project will guide the new NMCP strategic plan to be implemented in Mozambique until 2027. By demonstrating the benefits and complementarities of MMS to standard surveillance approaches, we hope to raise awareness of the potential of genomic science to guide interventions for effective malaria control and elimination. By streamlining sampling and molecular analysis to deliver MMS with speed, scale and quality, and by training junior researchers and decision-makers, we aim to increase the future sustainability of MMS in the country. Overall, the project will reduce the gap in the use of MMS data in Africa and increase the equitable access to technological platforms such as high-throughput sequencing to improve the health of the world’s population.

## supplementary material

10.1136/bmjopen-2024-092590 online supplemental file 1
