# Supplementary material for: Plasmodium falciparum molecular surveillance to inform the Mozambican National Malaria Control Programme strategy: protocol
Source: BMJ Open. 2024 Nov 24;14(11):e092590. doi: 10.1136/bmjopen-2024-092590 (PMC11590854; doi:10.1136/bmjopen-2024-092590)
Supplement: online supplemental file 1 [file bmjopen-14-11-s001.doc]

**Annex 1.** Brochures produced between 2021 and 2024 for dissemination of the malaria molecular surveillance activities to national stakeholders.

**A)** Brochure 1. Molecular markers of antimalarial resistance, 2018.


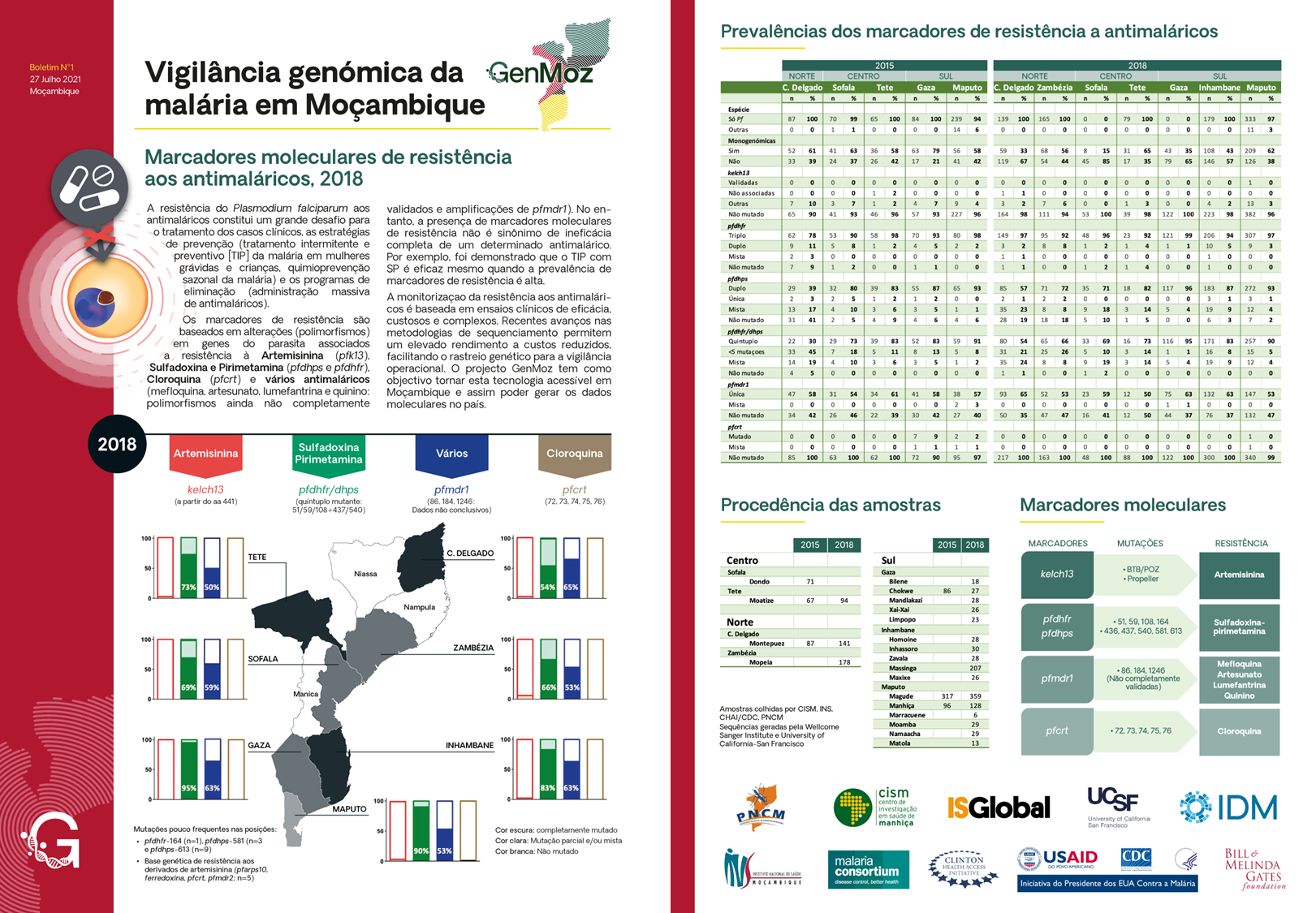


**B)** Brochure 2. GenMoz project.


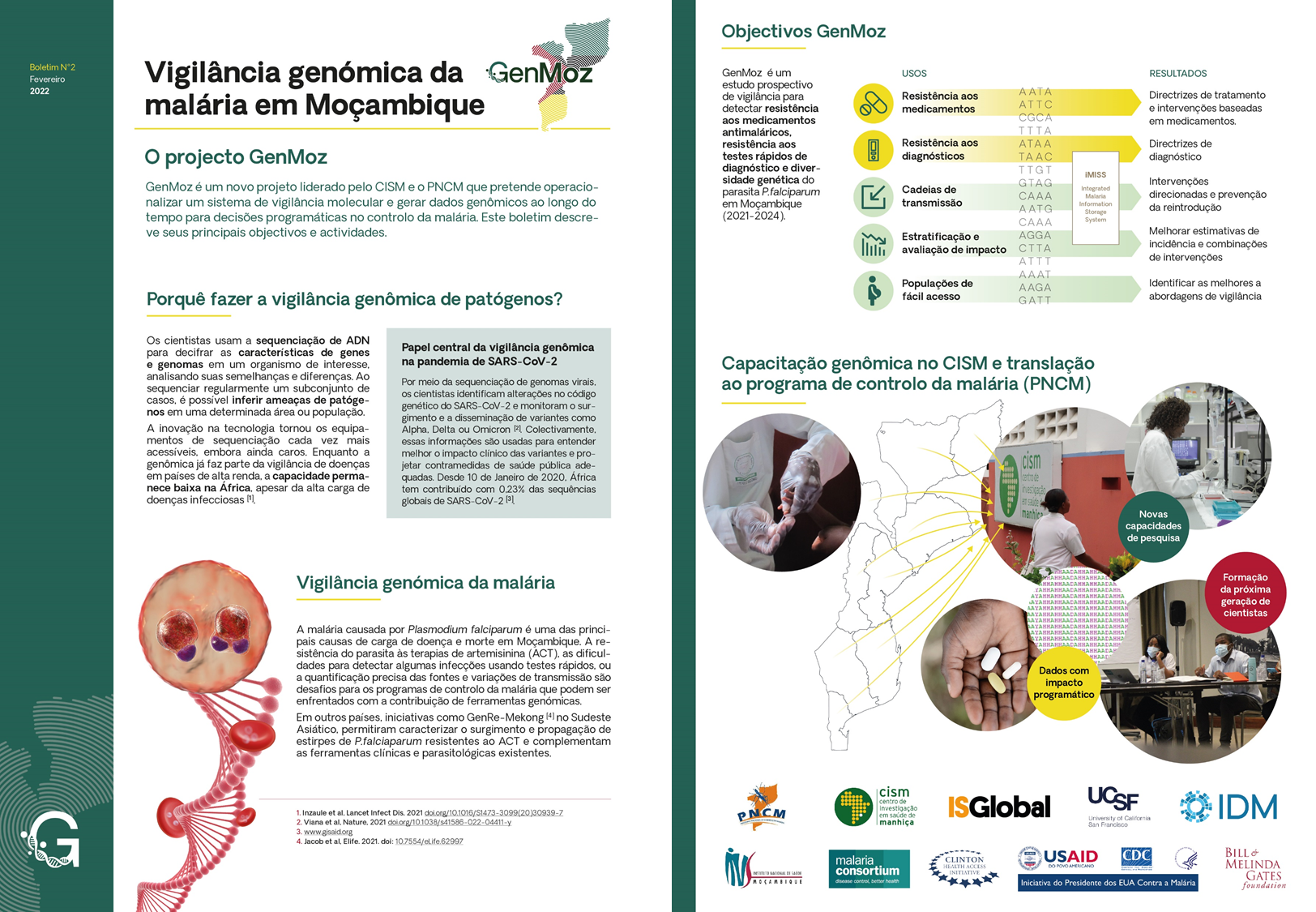


**C)** Brochure 3. Malaria sampling strategies.


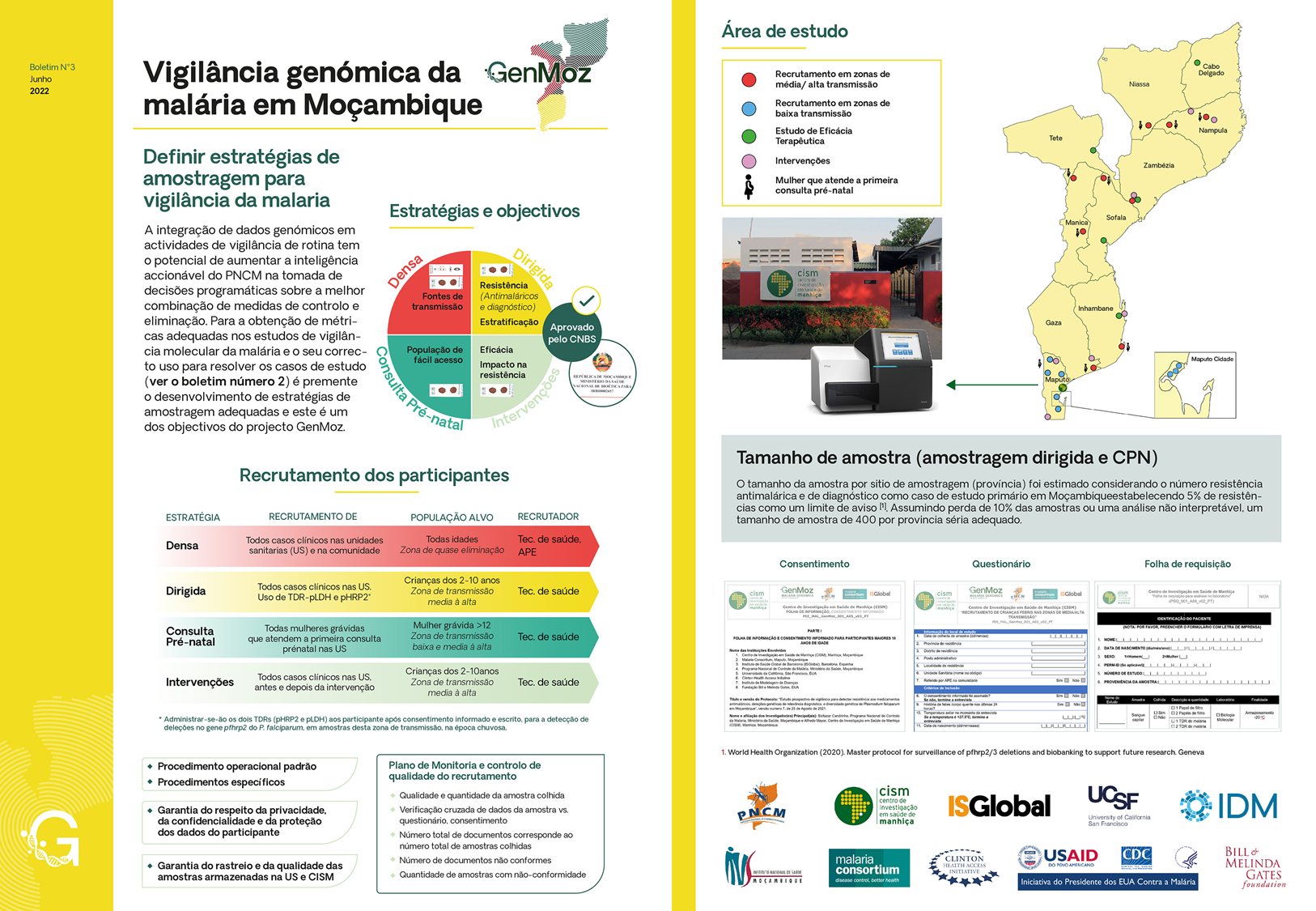


**D)** Brochure 4. Generation of genomic data in the laboratory.


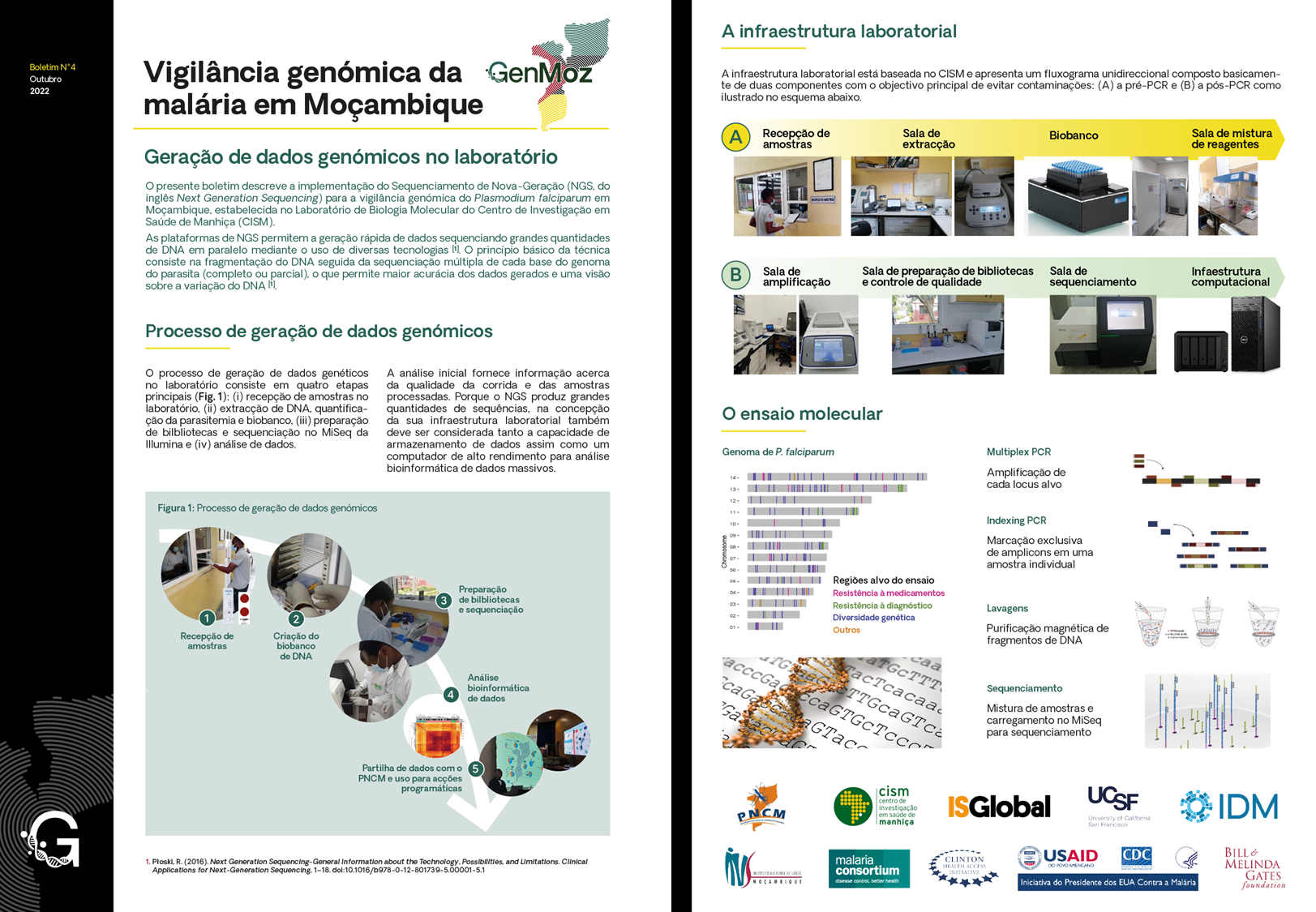


**E)** Brochure 5. Bioinformatic analysis of genomic data.


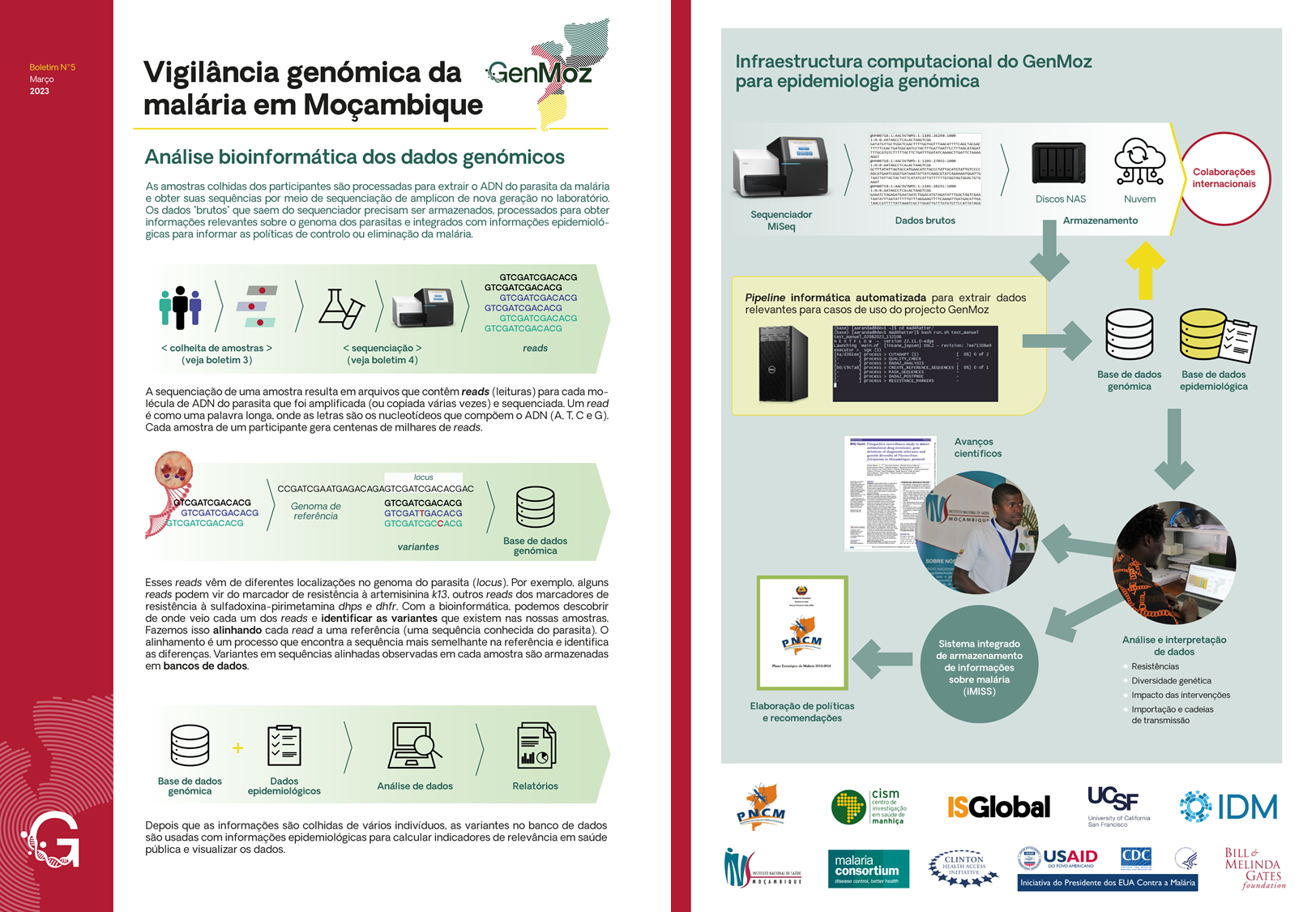


**F)** Brochure 6. Epidemiological models to integrate malaria molecular surveillance into programmatic decision making.


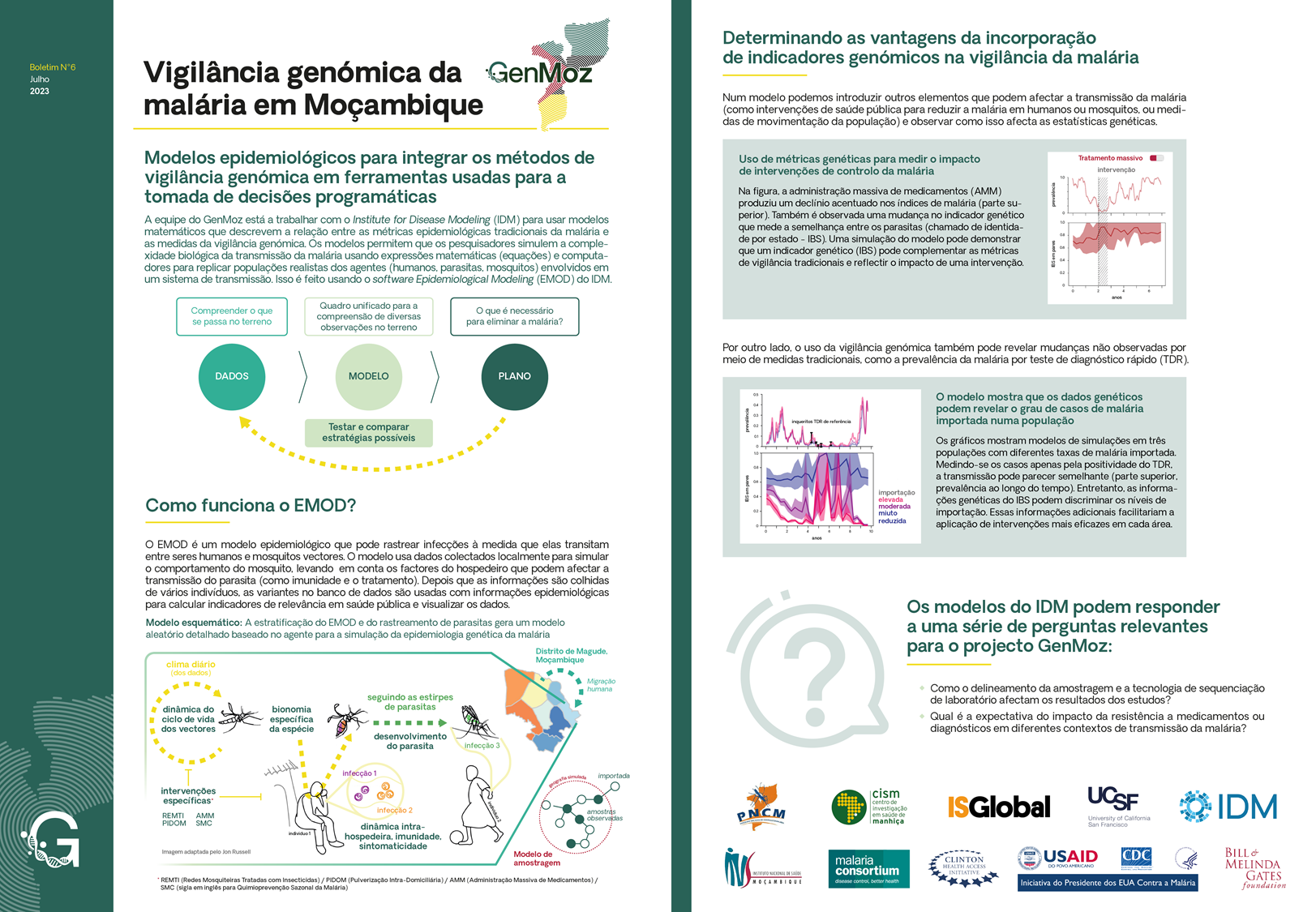


**G)** Brochure 7. Use of genetic information to identify the origin of infections.


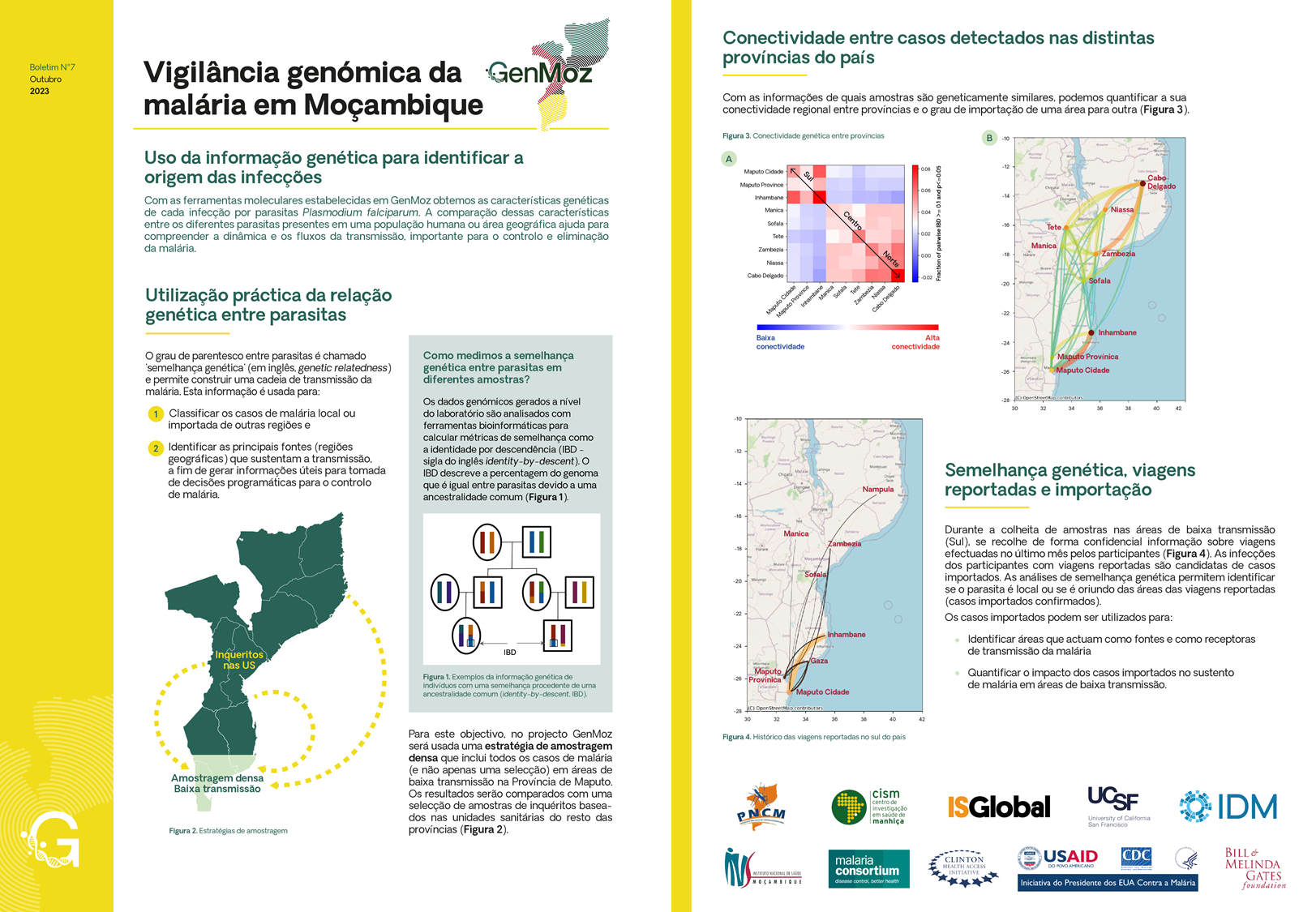


**H)** Brochure 8. Malaria genetic panels for monitoring and decision making.


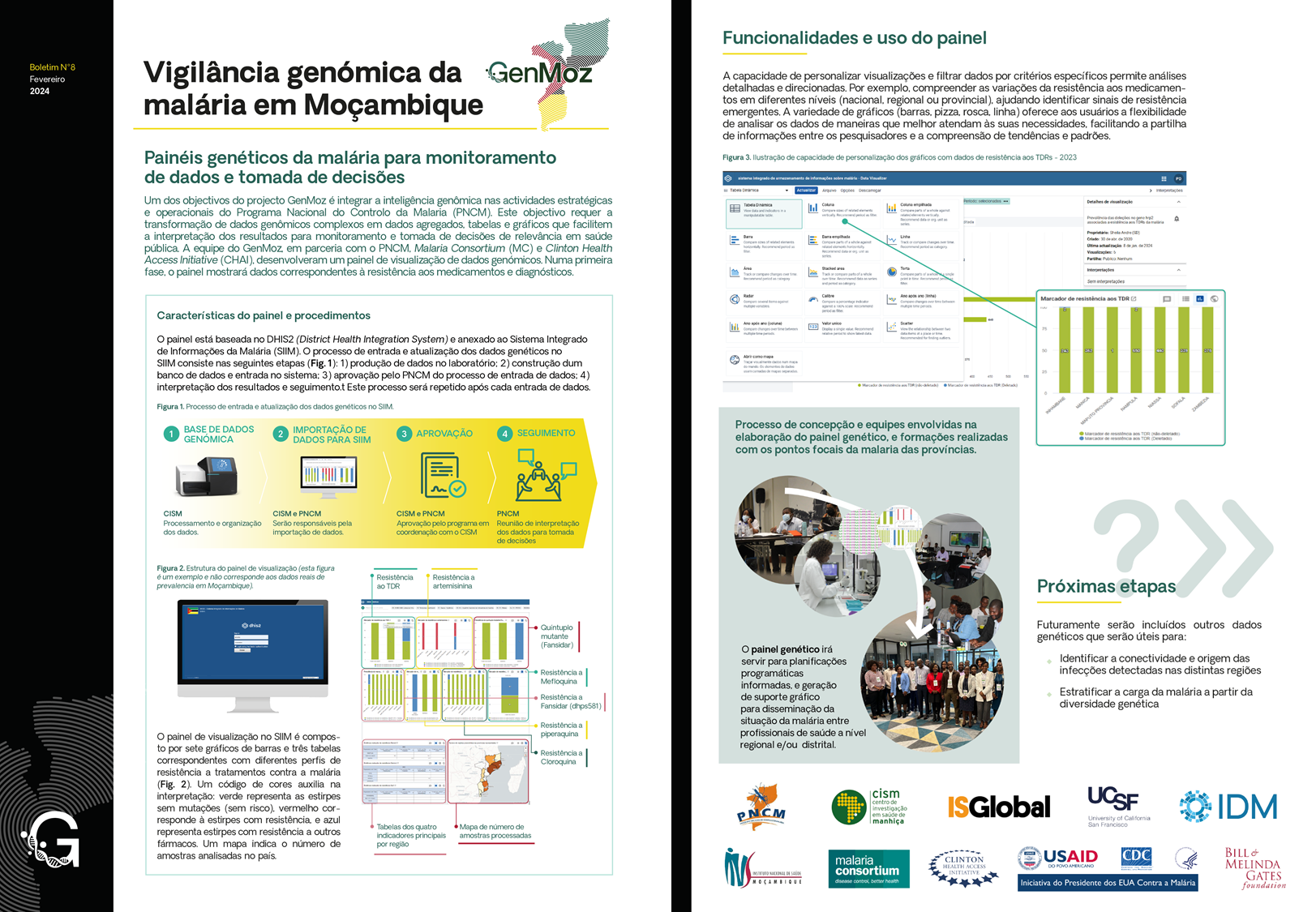


**I)** Brochure 9. Pregnant women at first antenatal care visit as sentinel group.

**
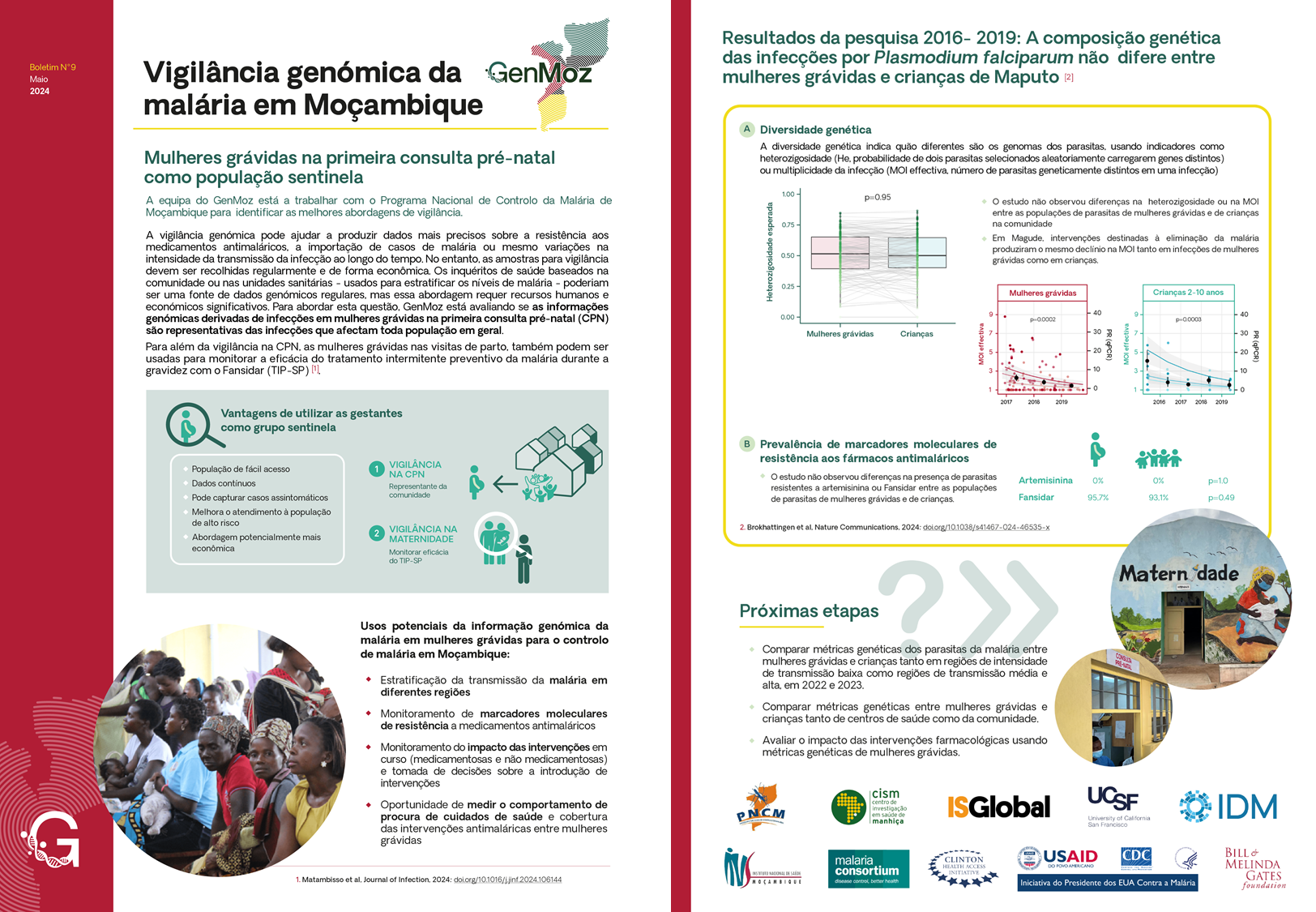
**
