# Supplementary material for: Plasmodium falciparum molecular surveillance to inform the Mozambican National Malaria Control Programme strategy: protocol
Source: BMJ Open. 2024 Nov 24;14(11):e092590. doi: 10.1136/bmjopen-2024-092590 (PMC11590854; doi:10.1136/bmjopen-2024-092590)
Supplement: Supplementary file 2 [file bmjopen-2024-092590.reviewer_comments.pdf]

PEER REVIEW HISTORY

BMJ Open publishes all reviews undertaken for accepted manuscripts. Reviewers are asked to complete a checklist review form and are provided with free text boxes to elaborate on their assessment. These free text comments are reproduced below.

ARTICLE DETAILS

Title (Provisional)

Plasmodium falciparum molecular surveillance to inform the Mozambican National Malaria Control Program strategy: Protocol

Authors

da Silva, Clemente; Matambisso, Gloria; Boene, Simone; Rovira-Vallbona, Eduard; Pujol, Arnau; Comiche, Kiba; Sánchez, Antoni; Greenhouse, Bryan; Chidimatembue, Arlindo; Aranda-Díaz, Andrés; Arnaldo, Paulo; Ariani, Cristina; Walker, Patrick; Mbeve, Henriques; Ndimande, Nelo; Tembisse, Dário; Ruybal-Pesántez, Shazia; Verity, Robert; Rafael, Bernardete; Candrinho, Baltazar; Mayor, Alfredo

This article was not externally reviewed at BMJ Open. Protocols that have been independently assessed prior to submission to BMJ Open are usually fast-tracked to publication on the grounds that further substantial changes will not be possible. This independent assessment will usually be external, independent review for both a substantial grant award from a non-commercial or government funder and ethics approval.
